# The search for life signatures on Mars by the Tianwen-3 Mars sample return mission

**DOI:** 10.1093/nsr/nwae313

**Published:** 2024-10-21

**Authors:** Zengqian Hou, Jizhong Liu, Yigang Xu, Fuchuan Pang, Yuming Wang, Liping Qin, Yang Liu, Yu-Yan Sara Zhao, Guangfei Wei, Mengjiao Xu, Kun Jiang, Chuanpeng Hao, Shichao Ji, Renzhi Zhu, Bingkun Yu, Jia Liu, Zhenfeng Sheng, Juntao Wang, Chaolin Zhang, Yiliang Li

**Affiliations:** Deep Space Exploration Laboratory, China; Institute of Geology, Chinese Academy of Geological Sciences, China; Deep Space Exploration Laboratory, China; Lunar Exploration and Space Engineering Center, China National Space Administration, China; State Key Laboratory of Isotope Geochemistry, Guangzhou Institute of Geochemistry, Chinese Academy of Sciences, China; Deep Space Exploration Laboratory, China; Lunar Exploration and Space Engineering Center, China National Space Administration, China; Deep Space Exploration Laboratory, China; School of Earth and Space Sciences, University of Science and Technology of China, China; Deep Space Exploration Laboratory, China; School of Earth and Space Sciences, University of Science and Technology of China, China; State Key Laboratory of Space Weather, National Space Science Center, Chinese Academy of Sciences, China; Research Center for Planetary Science, College of Earth Science, Chengdu University of Technology, China; Deep Space Exploration Laboratory, China; Deep Space Exploration Laboratory, China; Deep Space Exploration Laboratory, China; Deep Space Exploration Laboratory, China; Deep Space Exploration Laboratory, China; Deep Space Exploration Laboratory, China; Deep Space Exploration Laboratory, China; Deep Space Exploration Laboratory, China; Deep Space Exploration Laboratory, China; Deep Space Exploration Laboratory, China; State Key Laboratory of Space Weather, National Space Science Center, Chinese Academy of Sciences, China; University of Chinese Academy of Sciences, China; Department of Earth Sciences, The University of Hong Kong, China

## Abstract

We present the proposed strategic study, ‘Integrated elements for Martian life signature exploration’, to support the sampling and identification of any potential biosignatures in compliance with the engineering constraints of the Tianwen-3 mission.

Mars may have had a similar evolutionary history to Earth, which has inspired the search for potential biosignatures on Mars for decades. Particularly, there is an urgent need for a Mars sample return mission. China's Tianwen-3 (TW-3) mission has been planned to return Martian samples, the primary scientific goal of which is to search for signatures of life on Mars. Here, we present the proposed strategic study, ‘integrated elements for Martian life signature exploration’, to support the sampling and identification of any potential biosignatures in compliance with the engineering constraints of the TW-3 mission. The integrated elements for biosignature exploration of the strategy include ‘where to sample’, ‘what to choose’, ‘how to sample’, and ‘how to utilize’.

‘Where to sample’ refers to selecting landing sites with high potential for biosignatures. This involves identifying potential landing areas that are conducive to the development of habitable environments and the preservation of biosignatures. From an engineering perspective, it is necessary to choose areas where the lander can safely land, by taking into account latitude, altitude, terrain topography, rock abundance, and other constraints. From a scientific perspective, factors such as geologicalcontext, habitable environments, and geological diversity need to be considered [1]. Specifically, four aspects are considered in this perspective. (1) The exploration of habitable environments, which are suitable for the origin and survival of life, and spatial scales can range from planetary systems to microscale environments suitable for microbial survival [2]. Specific studies may focus on the formation mechanisms of water-related landforms and the coupling of geological evolution and life factors in active regions with an energy supply. (2) The study of habitable geological types should be conducive to preserving ancient life characteristics, such as deltaic, lacustrine, or oceanic environments. (3) To understand water evolution from different hydrated minerals with an indication of different temperature and pressure conditions, acidities or alkalinities, and water:rock ratios, providing important information for understanding the history of water activity and evaluating the potential preservation of biological signals. Regions enriched with hydrated minerals are promising areas for identifying ancient biosignatures and researching Martian paleoclimates and paleoenvironments. (4) A comprehensive evaluation of landing site selection based on multipleconstraint analyses, such as scientific constraints on life-sustaining hydrated minerals and water-related landforms and engineering constraints on slope, rock abundance, radiant conditions, and atmospheric activity, is performed. A criterion is established which supports the selection of landing sites that are both safe for engineering constraints and of high scientific merit.

The engineering constraints on the TW-3 landing site should consider altitudes ≤ - 3 km, latitudes ranging from 17° to 30°N, slopes ≤8°, and rock abundances ≤10%. Other engineering constraints, such as dust storm conditions, illumination, and temperature also need to be considered further. Currently, the TW-3 science group proposes 86 potential landing sites, primarily concentrated in the Chryse Planitia region and Utopia Planitia region (Fig. 1). These sites encompass diverse geological environments, such as ancient coastlines, deltas, ancient lakes, and canyon systems, providing favorable conditions for the origin and preservation of ancient life [3].

**Figure 1.**
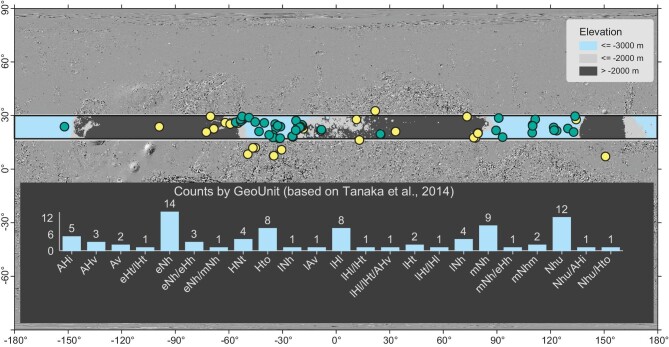
Geographical position and geological units of the preliminary landing sites (for the geological time of the stratigraphic unit, refer to [4]). Both the topographic shade map and elevation of potential landing sites are derived from the blended DEM of Mars MGS MOLA and MEX HRSC with a resolution of 200 m/pixel. The 51 green dots indicate the potential landing sites that comply with the current engineering constraints. The remaining 35 yellow dots do not meet the current engineering constraints but have relatively high scientific merits. The latitudinal zone between the two bold lines indicates the engineering constraint for 17°–30°N. The geological unit name of each label is presented here for more readability: AHi, Amazonian and Hesperian impact; AHv, Amazonian and Hesperian volcanic; Av, Amazonian volcanic; eHt, early Hesperian transition; lHt, late Hesperian transition; eNh, early Noachian highland; eHh, early Hesperian highland; mNh, middle Noachian highland; HNt, Hesperian and Noachian transition; Hto, Hesperian transition outflow; lNh, late Noachina highland; lAv, late Amazonian volcanic; lHl, late Hesperian lowland; mNhm, middle Noachina highland massif; Nhu, Noachian highland undivided.

‘What to choose’ focuses on how to identify, where to find, and how to preserve biosignatures, and systematically establishing identification methods to differentiate potential biosignatures, false positives, and false negatives. This research involves mainly Mars analog studies on Earth environments or laboratory environments characterized by extreme aridity, intense UV radiation, and an extremely low probability of microbe survival [5]. These investigations involve: (1) biosignature preservation carriers and their capabilities, such as potential biosignatures that have strong preservation capabilities or that exist in other forms and biosignatures that have the potential to be observed or detected [6–8]; (2) search for biosignatures in Mars analog environments and study changes in microbial features in terrestrial samples exposed to simulated Mars environments. Establishing Mars environment simulation laboratories to simulate specific parameters of different geological periods on Mars, and then studying the preservation capabilities ofpotential biosignatures in different rocks and minerals [9], which can provide preferred mineral types for sampling.

‘How to sample’ defines the depth and grain size requirements for Mars sample collection. For the returned samples, there will be noticeable differences in the types and characteristics of the samples required for mineralogical and biomaterial analysis. Mineralogical analysis requires block samples with geological diversity, while biosignature analysis requires loose clay minerals or sedimentary rocks. Additionally, modern Mars has a thin atmosphere and lacks global magnetic field protection, resulting in extreme environmental conditions such as solar and cosmic radiation and oxidation. To collect samples containing high-potential biosignatures, research on the following two aspects needs to be conducted: (1) surface sampling strategies considering the mineralogical characteristics of different types of samples,optimizing the particle size requirements, sampling sieve mesh sizes, and proportions of fine powder samples, rock fragments, and rock samples to support the development of surface sampling and drone assistance sampling strategies that meet scientific goals. (2) Drilling sampling strategies considering the influence of surface oxidation conditions, weathering, and particle radiation characteristics at different depths of regolith and/or sedimentary rocks on potential buried organic matter; a reference can be provided for selecting the appropriate sampling depth. To accurately and timely detect obstacles encountered during drilling, such as rocks and bedrock, research on regolith profile drilling and inversion of physical parameters is also needed. It is worth noting that the sampling sites including surface sample collection and drilling must be prevented from contamination during the TW-3’s *in situ* sampling.

‘How to utilize’ emphasizes that the returned sample should be stored following a planetary protection strategy before laboratory analysis. Currently, the biosignatures used in the detection of extraterrestrial life mainly include living organisms, fossils, substances derived from life processes, and chemical signals [10]. The interpretation of biological origin-related features often requires observations based on multiple complementary biosignatures. In addition, it is also essential to understand which biosignatures can be preserved in the Martian environment and how the environment influences these biosignatures, which may play a crucial role in establishing detection methods. The detection of biosignatures will require an increasing number of instruments specifically designed for astrobiological objectives, such as micro- and macro-imaging, spectral imaging, mass spectrometry analysis, and nucleotide sequencing. Three main aspects need to be considered: (1) the study of living organisms and the study of biosignatures. Research on low–molecular-weight metabolites in microbial cells or extracellularly indicates a Martian-like environment. Magnetic resonance imaging and mass spectrometry, along with high-throughput separation and data processing tools, are used to identify and quantitatively analyze microbial metabolites, assess the complexity and diversity of different metabolites, determine unknown metabolites, and analyze and interpret the data. Additionally, spectroscopic characterization of different organic molecules is performed to establish spectral identification methods and databases for identifying the biological features of organic molecules. Comprehensive research on the soluble and insoluble organic compounds of samples, such as Martian meteorites and surficial regolith, is undertaken to clarify the types, sources, and formation processes of organic compounds in Martian samples and to effectively distinguish potential biosignatures, false positives, and false negatives. (2) Identification and analysis of life-signifying minerals and microscopic fossils. Microbial processes can induce the formation of minerals and distinctive sedimentary landforms. Because of the low temperatures during precipitation or mineralization and the influence of bioorganic molecules, biogenic minerals are typically characterized by amorphous or low-crystallinity structures. Integrated laboratory analysis, including physical, geochemical, biological, and mineralogical experiments, should be conducted on samples from various Martian environments to identify minerals formed through biological processes and to study the preservation features of microbial rocks. Furthermore, the geochemical behavior of life-related elements and the associated mineral products, as well as the long-term preservation potential of commonly occurring minerals and their combinations on the Martian surface as life signals (microbes, body fossils, trace fossils, chemical fossils, etc.), need to be investigated. (3) Quantitative analysis of elemental and isotopic biosignatures. Focusing on life-related elements and isotopes, quantitative analysis methods need to be developed based on the physical and chemical properties of each element to identify the chemical forms of nonmetal elements in samples and deduce and interpret habitability and life materials. Additionally, the isotopic fractionation of life-related elements generated through bio-metabolism has been studied as a means to identify biosignatures. Experimental simulations are employed to investigate the biological fractionation of isotopes in the Martian environment, presenting an effective approach to search for life remnants in Martian returned samples. For example, laboratory simulations of microbial activity have been conducted to explore the S, Ni, and Mo isotopic fractionation characteristics induced by bio-metabolism in both Earth and Martian environments and to develop methods for the identification of isotopic fractionation biosignatures.

The TW-3 mission has set the exploration of potential biosignatures as a primary scientific objective and systematic research framework of ‘where to collect’, ‘what to choose’, ‘how to sample’, and ‘how to utilize’. Through integrated methods, including remote sensing and *in situ* data interpretation, comparative studies with Mars-like environments, laboratory simulations of Martian conditions, meteorites, and simulated samples, detailed research has been conducted on the types of potential biosignatures, reservoirs, sample collection strategies, and detection and analysis methods. This study will effectively support the TW-3 mission in achieving significant discoveries in the exploration of biosignatures on Mars, and contribute to the establishment of a scientific theoretical framework for the origin and evolution of life.
